# List of Diseases in London

**Published:** 1814-07

**Authors:** 


According to the report of several *Apothecaries* residing in various districts of the metropolis, the following was the average of the diseases in London between April 20th and May 19th:—

| | | | | | |
|---|---|---|---|---|---|
| Anasarca | 30 | Enterodynia | 20 | Pemphigus | 1 |
| Ascites | 4 | Enuresis | 1 | Paralysis | 24 |
| Asthma | 76 | Epilepsia | 13 | Phthisis Pulmonalis | 36 |
| Acne | 5 | Epistaxis | 2 | Pertussis | 44 |
| Apoplexia | 10 | Febris remittens | 103 | Peritonitis | 14 |
| Aphtha | 7 | —— intermittens | 31 | Phrenitis | 7 |
| Amenorrhœa | 33 | —— puerpera | 9 | Pneumonia | 76 |
| Asthenia | 31 | Gastritis | 7 | Pleuritis | 12 |
| Abortio | 14 | Gastrodynia | 33 | Podagra | 19 |
| Atrophia | 2 | Hæmatemesis | 6 | Porrigo larvalis | 6 |
| Bronchitis acuta | 10 | Hæmoptoe | 13 | —— scutulata | 12 |
| —— chronica | 20 | Hæmorrhois | 32 | —— favosa | 2 |
| Cancer | 2 | Hepatitis | 43 | Psoriasis | 29 |
| Chlorosis | 23 | Hysteria | 27 | Purpura | 1 |
| Cholera | 20 | Hysteritis | 3 | Pyrosis | 11 |
| Chorea | 1 | Hydrocephalus | 13 | Prurigo | 1 |
| Cephalalgia | 56 | Hydrothorax | 6 | Rheumatis. acut. | 73 |
| Colica | 18 | Herpes | 20 | —— chron. | 106 |
| —— Pictonum | 1 | Hypochondriasis | 15 | Rubeola | 122 |
| Catarrhus | 254 | Icterus | 22 | Scabies | 74 |
| Convulsio | 20 | Impetigo | 4 | Scarlatina simplex | 24 |
| Carditis | 4 | Ischuria | 9 | —— anginosa | 26 |
| Cynanche Tonsillaris | 99 | Leucorrhœa | 28 | —— maligna | 6 |
| —— maligna | 4 | Lithiasis | 3 | Scrofula | 32 |
| —— Trachealis | 10 | Lepra | 3 | Synochus | 7 |
| —— parotidea | 6 | Lichen | 6 | Tabes Mesenterica | 14 |
| Diabetes | 3 | Lumbago | 1 | Typhus | 11 |
| Dysenteria | 18 | Mania | 10 | Variola | 27 |
| Diarrhœa | 110 | Menorrhagia | 57 | Varicella | 16 |
| Dyspnœa | 22 | Miliaria | 3 | Vermes | 32 |
| Dyspepsia | 126 | Morbi Infantiles | 161 | Vertigo | 37 |
| Dysuria | 5 | —— Biliosi | 149 | Urticaria | 26 |
| Ecthyma | 1 | Morbus Pediculosus | 1 | | |
| Eczema | 4 | Nephritis | 4 | | |
| Erythema | 3 | Obstipatio | 41 | Total | 2967 |
| Erysipelas | 37 | Odontalgia | 1 | | |
| Enteritis | 8 | Ophthalmia | 50 | | |

The Deaths in the Bills of Mortality from April 12th to May 10th, 1814, according to the returns of the *Parish Clerks*, were as under:—

| | |
|---|---|
| Males | 661 |
| Females | 617 |
| Total | 1278 |

LONDON

